# Keeping their distance? Odor response patterns along the concentration range

**DOI:** 10.3389/fnsys.2012.00071

**Published:** 2012-10-18

**Authors:** Martin Strauch, Mathias Ditzen, C. Giovanni Galizia

**Affiliations:** ^1^Department of Neurobiology, University of KonstanzKonstanz, Germany; ^2^Bioinformatics and Information Mining, University of KonstanzKonstanz, Germany

**Keywords:** calcium-imaging, honeybees, glomerular pattern, odor concentration coding, odor identity coding, chemical dissimilarity

## Abstract

We investigate the interplay of odor identity and concentration coding in the antennal lobe (AL) of the honeybee *Apis mellifera*. In this primary olfactory center of the honeybee brain, odors are encoded by the spatio-temporal response patterns of olfactory glomeruli. With rising odor concentration, further glomerular responses are recruited into the patterns, which affects distances between the patterns. Based on calcium-imaging recordings, we found that such pattern broadening renders distances between glomerular response patterns closer to chemical distances between the corresponding odor molecules. Our results offer an explanation for the honeybee's improved odor discrimination performance at higher odor concentrations.

## 1. Introduction

The nature of olfactory systems seems to inevitably entangle odor identity and concentration. Across species, the response patterns of olfactory glomeruli encode odor identity (Malnic et al., 1999; Galizia et al., 2000; Wang et al., 2003; Falasconi et al., 2012), and more glomeruli join the response patterns with increasing odor concentration (Malnic et al., 1999; Wachowiak et al., 2002; Sachse and Galizia, 2003; Wang et al., 2003), i.e., patterns, as well as distances between patterns change. Is this a bug or a feature?

The olfactory system should allow an animal to recognize odors (discrimination) both in the presence of small fluctuations in chemical composition (generalization to similar odors) and at different dilutions (concentration generalization). Thus, two desiderata for an olfactory system are that (1) distances between odor response patterns should be realistic estimates of the odors' chemical dissimilarity, and that (2) distances should remain stable across a range of odor concentrations. Can such requirements be met by glomerular response patterns that broaden with increasing concentration?

We address these questions by analyzing calcium-imaging data from the honeybee *Apis mellifera*. Honeybees are amenable to behavioral testing of odor perception and it is known that they can discriminate odors better when odor concentration is high (Pelz et al., 1997; Wright and Smith, 2004). Here we study the basis for this improved performance. Optical imaging with calcium-sensitive fluorescent dyes is an established technique (Galizia et al., 1999b; Sachse and Galizia, 2003) for recording responses of many olfactory glomeruli simultaneously, rendering the honeybee a suitable model organism.

In the honeybee, approximately 60,000 receptor-equipped olfactory sensory neurons (OSNs) (Esslen and Kaissling, 1976) on each antenna project onto the 160 glomeruli of the antennal lobe (AL) (Galizia et al., 1999a), the first odor processing center in the insect brain. Glomeruli integrate responses from OSNs of one type and relay the information to higher-order brain centers via ca. 800 (Galizia and Rössler, 2010) projection neurons (PNs), i.e., multiple PNs leave from one glomerulus. Up to 4000 interneurons (Witthöft, 1967) that innervate the glomeruli are present in the AL, performing tasks such as contrast enhancement (Sachse and Galizia, 2002). By staining PNs with the calcium-sensitive fluorescent dye Fura-2 dextran, we can gain access to the glomerular activity patterns as they occur at the AL output, i.e., after integration and preprocessing. In mammals, this would correspond to activity patterns across mitral and tufted cells in the olfactory bulb. Here, these patterns represent the input to higher brain centers, such as the mushroom body and the lateral protocerebrum.

Based on these glomerular patterns, we investigate the representation of odor identity and concentration in the honeybee brain, recording glomerular responses to 16 odors at four concentration levels. The panel of 16 odors contains two major axes of variation, carbon chain length (6, 7, 8, and 9 carbon atoms) and functional group (primary alcohol: ol1, secondary alcohol: ol2, aldehyde: al, and ketone: on). Combining these features gives rise to the 16 odors, e.g., 6ol1 denotes 1-hexanol. Odors were delivered at concentration levels (from lowest to highest) 10^−4^, 10^−3^, 10^−2^, and 10^−1^ dilution in the solvent mineral oil.

In particular, we focus on the dissimilarity of odor response patterns as measured by the Euclidean distances between them. Distances readily translate into relative positions of the odors in response pattern space, they allow us to assess how well response pattern space is aligned with chemical space, and they provide information about error tolerance and the margin of separation between “odor code words.”

In the following, we first draw a global picture of the honeybee's olfactory response space, visualizing distances between odors response patterns. We then portray how responses of individual glomeruli develop over the concentration range and analyze how these individual developments can be beneficial for distances between entire odor response patterns.

Our findings support the hypothesis that distance changes along the concentration range are not a bug, but a feature that improves representation of odor (dis)similarity in the brain. This extends prior work aimed at solving the problem of concentration-invariant perception (Asahina et al., 2009; Schmuker et al., 2011; Cleland et al., 2012) by emphasizing that there is also something to gain from increased odor concentration. Due to the similarities between olfactory systems, the hypothesis generalizes to other organisms and offers a straightforward explanation for why we can smell better when odor concentration is high.

## 2. Methods

### 2.1. Calcium-imaging

Calcium-imaging was performed as described in Ditzen (2005), where the present dataset was first published. Briefly, PNs in the AL of forager bees (*Apis mellifera*) were filled retrogradely with the calcium-sensitive fluorescent dye Fura-2 dextran. We used a TILL Photonics imaging system (TILL Photonics, Germany) consisting of an upright microscope (Olympus BX50WI, Olympus, Germany) equipped with a 20× objective (Olympus XLum PlanFl 20 ×/0.95W) and a CCD camera. Double images were recorded with 340 and 380 nm excitation light where the final signal was the ratio of the images taken at 340 and 380 nm.

Responses to the panel of 16 odors (see section 1) were recorded in distributed measurements in 53 bees. Each odor response measurement consisted of 40 double images recorded at a frequency of 5 Hz. Using a PAL system (CTC Analytics), the odor was always applied at the same time point during each measurement, lasting for 2 s. The odor consisted of 2 ml headspace from a 20 ml sealed glass vial filled with 5 ml diluted odor solution. Individual odor response measurements were separated by pauses of 2.5 min. Concentration series, i.e., sequences of the form “odor at concentration level 10^−4^ (dilution in mineral oil), at 10^−3^, at 10^−2^, at 10^−1^” were always measured blockwise in the same bee and in ascending order. Depending on the fitness of the bee, one or more such concentration series of different odors were measured, as well as multiple control measurements (air, mineral oil) and a reference odor, 1-nonanol (9ol1), that was measured in every bee. On average, bees were stimulated with 3.2 (± 1.2) different odors (including the reference odor). Only complete concentration series were used.

### 2.2. Signal extraction

**Figure 1:**
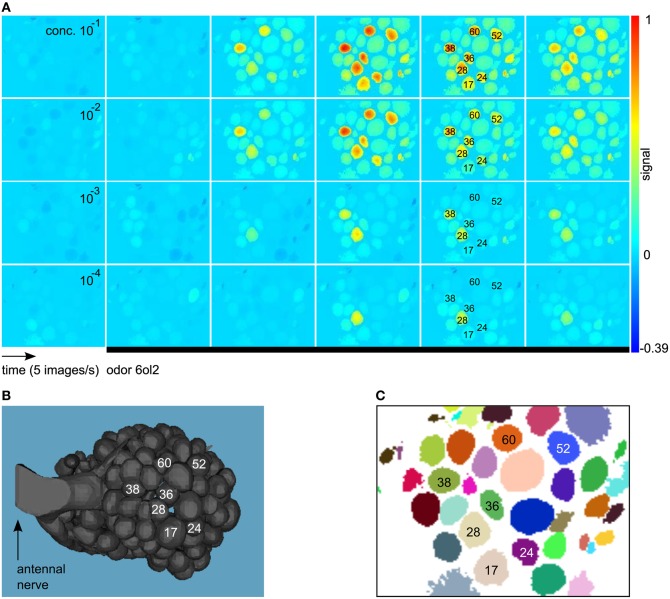
**(A)** Example for a calcium-imaging movie processed and visualized with the method from Strauch et al. (2012). We show consecutive images from the response to the odor 6ol2 at all four concentration levels. All images are false-color coded using the same min–max color scale. Shortly after odor application (black bar), some of the glomeruli increase their activity above baseline, giving rise to an odor-specific response pattern. Glomerulus numbers were assigned according to Galizia et al. (1999a). **(B)** Anatomical AL model modified from Brandt et al. (2005). Glomeruli with a prominent response to 6ol2 in **(A)** are marked. **(C)** Clustering that reveals positions of glomeruli (and other objects) in the movie from **(A)**.

Imaging movies were processed with the ImageBee plugin (http://tech.knime.org/imagebee-analysing-imaging-data-from-the-honeybee-brain) for the data analysis platform KNIME (KoNstanz Information MinEr, www.knime.org). In the processed movies (Figure 1A), glomerulus positions were clearly visible. Glomerulus identities could be inferred by visual inspection, matching an anatomical AL model with labeled glomeruli (Figure 1B) and a clustering of the imaging movie (Figure 1C).

For details on the methods employed in ImageBee, see our previous paper (Strauch et al., 2012). Briefly, imaging movies were corrected for animal movement by aligning consecutive measurements from the same bee. Then, glomerular signals were extracted based on a functional segmentation of the movie: pixels with the same signal in the time domain were assigned to the same cluster (Figure 1C). For each identifiable glomerulus in the clustering, the algorithm estimated the pure glomerular signal as the average of all time series within the glomerulus that are not contaminated by light scatter from neighboring glomeruli.

### 2.3. Data analysis

#### 2.3.1. Mean glomerular time series

All data analysis on extracted time series was carried out in R (R Development Core Team, 2011) For each glomerular time series, we subtracted the mean of the first 10 time points (before odor application) to obtain an odor response relative to baseline. All glomerular time series in one bee were scaled such that the highest response (maximum activity after odor stimulation) to the reference odor 9ol1 at 10^−1^ was set to 1.

From these standardized glomerular time series (in individual bees) we then computed the mean odor responses that were used for data analysis. Not all 64 odor/concentration conditions were measured in every bee. As odor responses are conserved across bees (Galizia et al., 1999b), we could average over all odor responses of the same glomerulus for the same odor/concentration condition. Mean responses were obtained from distributed measurements in 53 individual bees. We included all glomerulus/condition pairs into the analysis that were measured at least three times (μ = 6.06 times, σ = 2.44) in different bees.

All Figures are based on the mean responses, also referred to as “signal,” of the 20 glomeruli with sufficient count for all conditions. These are glomeruli from the frontal part of the AL that is accessible by optical imaging (cp. Figure 1B).

Note that not all of the 20 glomeruli were stained in every bee. Different sets of glomeruli were visible in different bees with an intersection too small for meaningful analysis of glomerular patterns. Instead, as stated above, we ensured that each glomerulus/condition pair was measured with sufficient count in different bees. The mean odor responses presented here are thus the responses of a “meta-animal.”

#### 2.3.2. Distance-preserving 2D space

**Figure 2:**
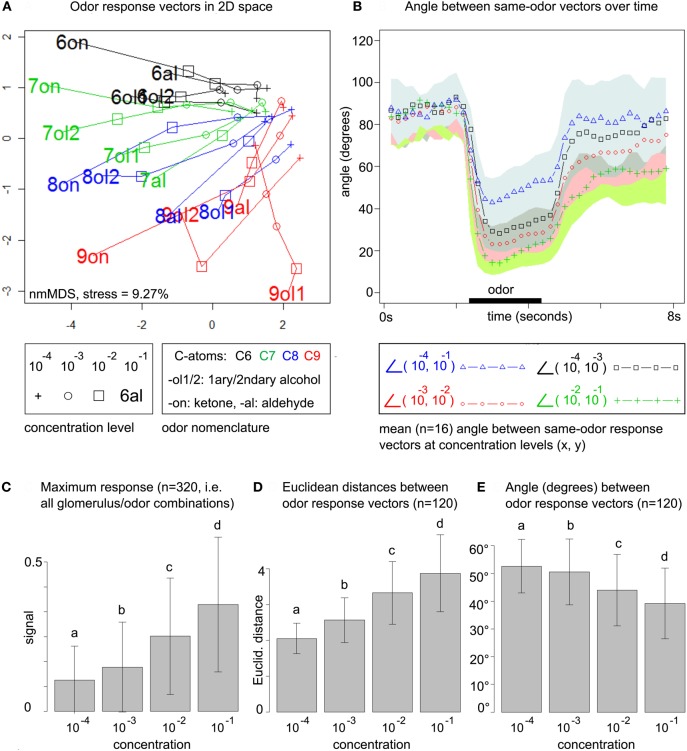
**(A)** Odor response patterns for the 16 odors at four concentration levels in a distance-preserving 2D space. Concentration levels of the same odor are connected by lines. See legend for details. Axes: arbitrary units. **(B)** Angle between response patterns (vectors) for the same odor at different concentrations. The blue line (triangles) indicates the mean angle (*n* = 16 odors) between same-odor response patterns at concentration levels 10^−4^ and 10^−1^ for each time point, i.e., before, during (black bar) and after odor presentation. The area between μ_*i*_ + σ_*i*_ and μ_*i*_ − σ_*i*_ is colored in light blue (mean μ and standard deviation σ for all time points *i*). Remaining lines: see legend. All colored areas (standard deviations) range from the lower bound (−σ_*i*_) up to the point where they overlap with the next area. Due to the large standard deviations such overlaps occur frequently. **(C)** Maximum response at the four concentration levels pooled over all 20 × 16 glomerulus/odor combinations. Bars indicate mean μ (of maximum responses) and standard deviation σ. All concentration levels are significantly different (repeated measures ANOVA, *F* = 441.73, *p* < 2.2 × 10^−16^, *post-hoc* testing with Holm-adjusted pairwise *t*-tests) **(D)** All (16 × (16 − 1))/2 pairwise Euclidean distances between the 16 odor response patterns at each of the four concentration levels. Bars indicate mean (of distances) and standard deviation. Same statistics as in **(C)**: *F* = 23.16, *p* = 5.28 × 10^−14^. **(E)** Pairwise angle between odor response pattern vectors. Same statistics as in **(C)**: *F* = 10.017, *p* = 2.068 × 10^−6^.

We employed Kruskal's non-metric multidimensional scaling [isoMDS, MASS-package (Venables and Ripley, 2002) ported for R] to compute distance-preserving embeddings in 2D space. Note that we use this technique only for visualization purposes and only in Figure 2A and in Figure 4B.

For computing the 2D space from Figure 2A, data was arranged in a matrix with stimulus conditions in the (concentrations × odors) rows and calcium responses in the (glomeruli × time points) columns. Euclidean distances between the 64 odor/concentration conditions in the rows of the matrix were computed based on the (glomeruli × time points) features in the columns, where we considered 20 time points (= 4 s) after odor stimulation. This approach conserves the full temporal complexity of the odor response, rather than compressing it (*a priori*) to the single maximum or mean odor response in each glomerulus. We then employed non-metric multidimensional scaling to compute the 2D space that optimally preserves Euclidean distances from the original, high-dimensional space.

Similarly, for the 2D space from Figure 4B data was arranged in a matrix with (concentrations × glomeruli) rows and (odors × time points) columns, and we computed an embedding of the 80 glomerulus/concentration combinations based on their odor response profiles.

The quality of the results can be evaluated by the so-called stress, a measure for the distortion of distances through dimensionality reduction. It is a dimensionless number usually given in percent, with 0% corresponding to perfect conservation of distances. According to Kruskal (1964), a stress of 5% is “good” and a stress of 10% is “fair.” Stress values for both figures are below 10%.

#### 2.3.3. Normalization

If (and only if) indicated in the text, we performed z-score normalization by subtracting the mean and dividing by the standard deviation. In particular, the odor response matrix *R* was partioned as

(1) $$R=\left(\begin{array}{c} C_{-4}\\ C_{-3}\\ C_{-2}\\ C_{-1} \end{array}\right)$$

Each block *C*_*i*_ was the odors × (glomeruli × time points) submatrix for the respective concentration level. Z-score normalization was performed separately on the features (columns) of each submatrix *C*_*i*_, such that each feature in each submatrix *C*_*i*_ had mean zero and standard deviation one.

This normalization removes the influence of (concentration-dependent) feature amplitude, and it adjusts the mean distances of the concentration groups. Both Euclidean distances (Figure 2D) and cosine distances (Figure 2E) lead to concentration groups that are significantly different with respect to the mean pairwise distance between odor response patterns. After z-score normalization, pairwise Euclidean distances are no longer significantly different between concentration groups (repeated measures ANOVA, *F* = 0.93 *p* = 0.43).

We employed z-score normalization as an analytical tool to compute distances independent of feature amplitude, and to compare concentration groups with different location/dispersion parameters. This allowed us to compute qualitative distance changes (Figure 5) between concentration groups with different mean distance, and it allowed us to analyze to which extent the correlation between response pattern distances and chemical distances (Figure 6) is due feature amplitude.

#### 2.3.4. Correlation with chemical distances

In order to measure chemical distances between odors, we employed a Euclidean distance metric for odor molecules that is based on selected and weighted chemical descriptors as features (Haddad et al., 2008). Descriptor values and weights were taken from the supplementary material of Haddad et al. (2008).

For the comparison of odor response pattern and chemical distances (Figure 6), we used the Mantel test (Mantel, 1967) from the ade4 package (Dray and Dufour, 2007) for *R*. The test statistic of the Mantel test is the correlation between the two distance matrices, and the significance of the correlation is assessed by a permutation analysis. *P*-values are based on 2000 permutations per time point and were corrected for multiple (4 × 40 time points) testing by Benjamini–Hochberg correction [Benjamini and Hochberg (1995), stats package for *R*] that controls the false discovery rate.

## 3. Results

### 3.1. A global view on ODOR response space

To obtain a global view on the honeybee's odor response space we arranged the data in an odor response matrix with stimulus conditions (concentrations × odor) as rows and calcium responses (glomeruli × time points) as columns. We embedded the 64 odor/concentration conditions in a 2D space (obtained by multidimensional scaling, see section 2) that preserves Euclidean distances between response patterns: Figure 2A.

This uncovers three main trends: (1) odor response space expands with rising concentration, i.e., Euclidean distances between patterns increase. (2) Pattern development along the concentration range is smooth, i.e., different concentrations of the same odor can be connected by straight lines (no zig-zag paths) in almost all cases. (3) odor response patterns are roughly sorted by chemical (dis)similarity of the odors, in particular carbon chain length.

These findings already provide us with first answers to the questions raised in the introduction. There seems to be an improvement (regarding pattern separation) with rising odor concentration (1), it appears possible to generalize over different concentration levels of the same odor (2), and response pattern (dis)similarities reflect chemical (dis)similarities (3). The remainder of this paper is dedicated to analyzing these aspects in greater detail.

We first quantify the smoothness of pattern development across concentrations. In Figure 2B, we plot the mean angle between response pattern vectors for the same odor at different concentration levels and for each time point during the measurements. We consider the angle between same-odor response patterns at concentration levels 10^−4^ and 10^−1^, i.e., for the entire concentration range. In addition, we also regard transitions between nearby concentration levels, such as 10^−4^ and 10^−3^. Mean angles are drawn as colored lines, while the standard deviation is indicated by the corresponding colored area.

Before odor stimulation, all angles are close to 90°. During odor stimulation, the angle between patterns at 10^−4^ and 10^−1^ decreases to a minimum of 43°, indicating that response pattern vectors change substantially with increasing concentration, but not arbitrarily, as they are still far from being orthogonal. The smoothness of the transition increases with concentration: while the lowest angle for the transition from 10^−4^ and 10^−3^ is 28.2°, it is 23.1° for the transition from 10^−3^ to 10^−2^, and 14.1° for the transition from 10^−2^ to 10^−1^.

From prior work on odor concentration coding in the honeybee AL (Sachse and Galizia, 2003) we know that increasing odor concentration leads to both an increase in the number of responding glomeruli and to a general increase in glomerular response amplitude. The latter can explain the expansion of odor response space in Figure 2A. Mathematically, if we scale the odor response matrix with a factor *s* > 1, the Frobenius norm of the induced Euclidean distance matrix will grow by that factor.

Indeed, our data supports prior findings (Sachse and Galizia, 2003), showing that maximum glomerular response pooled over all odor/glomerulus combinations increases over the concentration range (Figure 2C). As expected, this leads to an increase in pairwise Euclidean distances between odor response patterns (Figure 2D), simply because general response amplitude is elevated at higher odor concentrations. Standard deviations are high, but the trend reaches significance in stratified testing (stratified by odor pair, repeated measures ANOVA).

We also computed pairwise angles between odor response pattern vectors within the same concentration level: Figure 2E. Both, angles between same-odor vectors at different concentration levels (Figure 2B) and angles between different odor response vectors within the concentration levels (Figure 2E, not time-resolved) decrease with rising concentration. As even the lowest mean angle at 10^−1^ (between different odors, Figure 2E) is about 40°, this suggests that different-odor angles at the same concentration are larger than same-odor angles at different concentrations, at least for nearby concentration levels.

In summary, global statistics indicate an expansion of Euclidean distances with rising odor concentration, which can be seen as an improvement for reliable odor coding: responses higher above noise level are more robust, as are inflated distances between odor response patterns.

In contrast, angles (cosine distances) decrease with rising concentration, i.e., response patterns become, on average, more correlated. Unlike Euclidean distances, angles between vectors are not dependent on signal amplitude, which is why the general increase in response amplitude is not reflected in the angles. The decreasing angles can be explained by a larger number of responding glomeruli at high concentrations that leads to higher baseline correlation. This would indicate a non-linear development along the concentration axis with new glomerular responses joining the patterns, as opposed to a linear scaling of responses that were already present at the lowest concentration level. We will quantify the number of above-threshold responses in the next section.

So far, we can already conclude that changes between different concentration levels of the same odor are relatively smooth, providing the basis for generalization at least over nearby concentration levels.

### 3.2. Glomerular responses along the concentration axis

**Figure 3:**
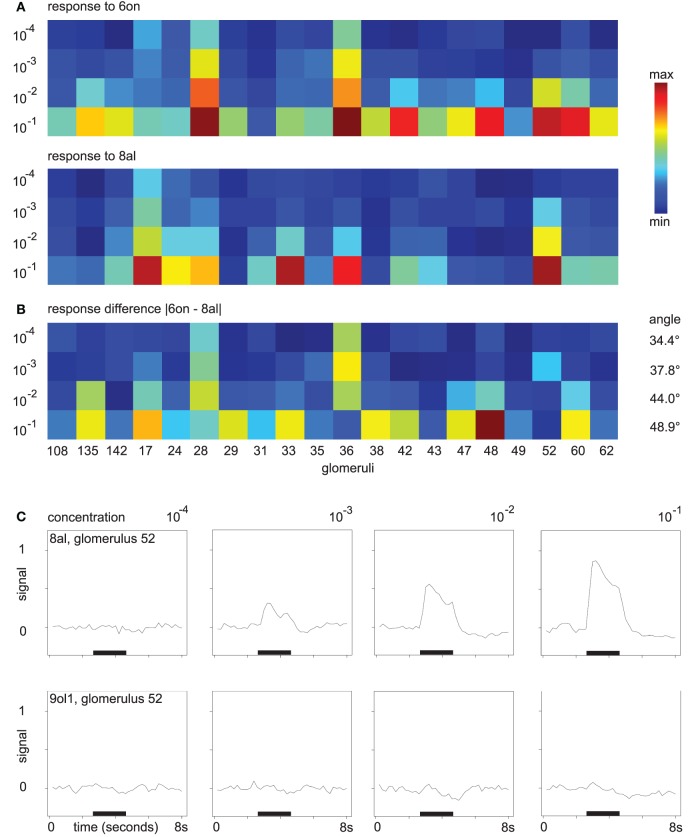
**(A)** Glomerular response pattern for the odors 8al and 6on, respectively, at all four concentration levels. Colors encode maximum response of the respective glomerulus. Both odors shown in the same min–max color scale with (min, max) = (0.0007, 0.9). **(B)** Difference |“response to 6on”–“response to 8al”|. Min–max color scale with (min, max) = (0.0004, 0.68). Angles between the vectors for 8al and 6on are annotated. **(C)** Mean time series of glomerulus 52 in response to the odors 8al and 9ol1. For all four concentration levels, odors were applied during the interval marked with the black bar.

As an example for odor responses along the concentration axis, consider the mean responses to the odors 6on and 8al. In Figure 3A, colors correspond to the maximum response of the respective glomerulus to four different concentrations of 8al or 6on, respectively. In Figure 3B, we also visualize distance development. Here, colors correspond to the difference in maximum response to the two odors 6on and 8al at each of the four concentration levels.

While amplitude and number of discriminatory glomerular responses are small at concentration level 10^−4^, both odors can be distinguished based on their response profiles. At concentration level 10^−4^, glomeruli 28 and 36 respond to 6on, but not to 8al. With increasing odor concentration, response amplitude increases and more glomeruli join the patterns. While the “sensitive responders” 28 and 36 are still prominent features of the response to 6on at concentration level 10^−1^, they now also participate in the response to the odor 8al, although with slightly lower response amplitude. On the other hand, new discriminatory features occur, e.g., glomerulus 48.

Note also, that, despite the general decrease in between-odor angles (Figure 2E), the angles between the odor response vectors in this example do in fact increase with rising concentration (Figure 3B), indicating that changes in odor dissimilarity or distance can be specific to the odor pair involved.

As a further example, Figure 3C shows the gradual buildup of an odor response (glomerulus 52 to the odor 8al), starting at concentration level 10^−3^. At the lowest odor concentration, glomerulus 52 has no discriminative power regarding the odors 8al and 9ol1, however, it builds up a response to 8al over the concentration range, while it does still not respond to 9ol1 at 10^−1^ (Figure 3C). Thus, the additional glomerular responses at higher odor concentrations contribute more information about the odor.

**Figure 4:**
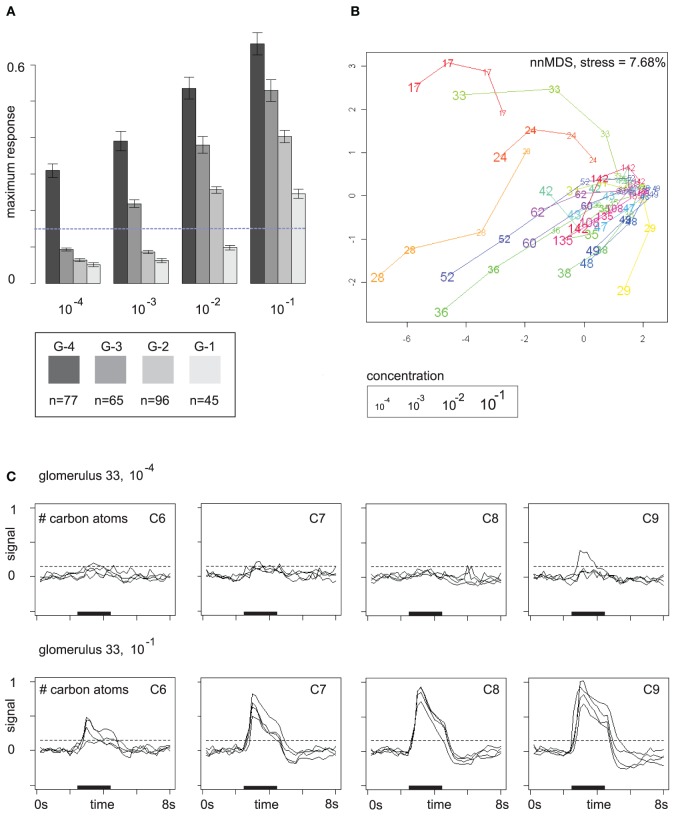
**(A)** Mutually exclusive glomerular response groups over the concentration range. Group G-4 contains all glomeruli that respond for the first time at concentration level 10^−4^, G-3 contains all glomeruli that respond for the first time at 10^−3^, etc. Bars show mean and standard error of the feature “maximum response.” The response threshold (dashed line) was set to 0.15. Glomeruli may exhibit sub-threshold responses at lower concentrations. **(B)** Glomerular response profiles at the four concentration levels in a distance-preserving 2D space. Axes: arbitrary units. **(C)** Response profile (all 16 odors) of glomerulus 33 at concentration levels 10^−4^ and 10^−1^ stratified by carbon chain length. Dashed lines mark the response threshold used for defining the groups in **(A)**.

The response buildup over the concentration range as shown in Figure 3C is in fact a general phenomenon. Glomeruli have a response threshold, a concentration level at which they respond to a particular odor for the first time, and then they increase their response to the odor when concentration rises further. Figure 4A summarizes the trend: here, glomerular responses (pooled over all odors) are grouped by the concentration level at which they respond to the respective odor for the first time. Group G-4 contains all glomerular responses that occur for the first time at concentration level 10^−4^, group G-3 contains all glomerular responses at concentration level 10^−3^ that did not yet occur at 10^−4^, etc.

Figure 4A reveals that patterns are broadened by additional glomerular responses at higher concentrations. In particular, gomeruli that responded already at low odor concentrations still respond at higher concentrations. On average, they also have stronger responses than glomeruli that joined the patterns later on (Figure 4A), which provides the basis for generalization over concentration levels by normalization or correction for the general response level at the respective odor concentration.

Glomerular response profiles are individual, but it may require high odor concentrations to be able to distinguish between them. Figure 4B visualizes Euclidean distances between glomerular odor response profiles at the four concentration levels by embedding them in a 2D space, again obtained by multidimensional scaling. Most glomeruli cluster densely together at the lowest odor concentration 10^−4^ as they exhibit no pronounced response to any odor, but then develop into different directions of response profile space when odor concentration is increased. Few “sensitive responders,” such as glomeruli 17 and 28, are already responsive to a broad spectrum of odors at low odor concentrations and are therefore clearly separated from the others.

An example for an odor response profile is given in Figure 4C: glomerulus 33 exhibits almost no odor responses at concentration level 10^−4^, but at 10^−1^ it has developed a broad response profile that provides information about carbon chain length as responses are stronger for odor molecules with long carbon chains. In the 2D space from Figure 4B, this development of glomerulus 33 over the concentration range is reflected by a long trajectory that starts within the dense cluster of non-responding glomeruli but then moves quickly outwards as the glomerulus develops its response profile.

Note that the value chosen for the response threshold determines group sizes in Figure 4A: for a threshold of 0.15 (with 1 being the maximum response to the reference odor), group sizes are (G-4, G-3, G-2, G-1) = (77, 65, 96, 45). This corresponds to the visual impression of a weak response in Figure 4C (see dashed lines). Doubling the threshold to 0.3 would lead to group sizes (G-4, G-3, G-2, G-1) = (30, 23, 69, 85). If we count only strong responses, that exceed the 0.3 threshold, the peak of glomerulus recruitment occurs at 10^−1^, whereas for the lower threshold 0.15 the peak occurs at 10^−2^. Thus, the pool of glomeruli that can be recruited begins to drain at about 10^−2^, but there are still glomeruli that can be recruited, and, moreover, response strength can still be increased.

Extrapolating this trend to higher (and possibly unnaturally high) odor intensities would cause saturation by too many, unspecific responses. However, for the concentration range tested here, additional glomerular responses at higher odor concentrations can contribute more information about the odor. In the next section, we investigate how response properties of individual glomeruli act together and adjust distances between entire odor response patterns along the concentration axis.

### 3.3. The nature of distance changes

We next asked how distance changes along the concentration axis affect the representation of chemical (dis)similarity in response pattern space. To this end, we employed a distance metric for odor molecules (see section 2), giving rise to chemical distances *d*(X,Y)_chemical_ between two odors X and Y.

**Figure 5:**
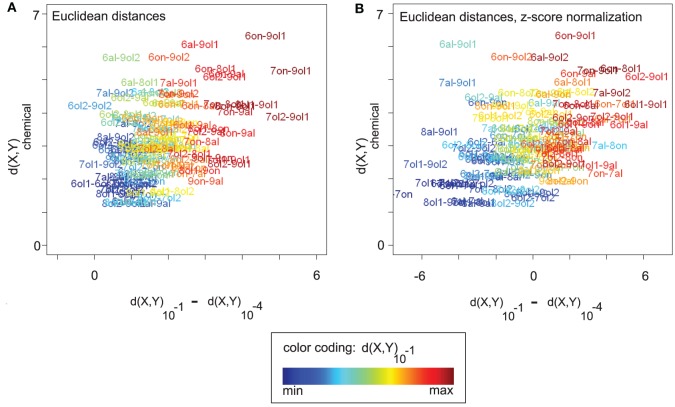
**(A)** All (16 × (16 − 1))/2 pairwise chemical distances *d*(X,Y)_chemical_ between odors X and Y vs. the corresponding changes in response pattern distance along the concentration axis, *d*(X,Y)_10^−1^_ − *d*(X,Y)_10^−4^_. Odor pairs are colored according to their response pattern distance at 10^−1^ (see legend). Odor response pattern distances are Euclidean distances. Changes in response pattern distance are correlated with chemical distances (see main text). **(B)** Same plot as in **(A)**, but data was z-score normalized (see section 2). After normalization, positive values on the x-axis correspond to larger-than-average distance changes (and negative values to lower-than-average changes). Qualitatively, the correlation between response pattern distance changes and chemical distances remains unaltered (see main text). Note that a purely linear scaling of distances from 10^−4^ could also explain the correlation in **(A)**. However, it would be removed by z-score normalization, as would the correlation.

Figure 5A visualizes the relationship between three measures, (1) the chemical distance *d*(X,Y)_chemical_, (2) the change in Euclidean distance between the corresponding odor response patterns along the concentration axis, *d*(X,Y)_10^−1^_ − *d*(X,Y)_10^−4^_, and (3) the distance *d*(X,Y)_10^−1^_ between the odor response patterns at the highest concentration level. Distance changes with rising concentration (x-axis) are correlated with both chemical distances (y-axis, Spearman's ρ = 0.38, *p* = 2.797 × 10^−5^) and distances at concentration level 10^−1^ (“color axis,” ρ = 0.87, *p* < 2.2× 10^−16^).

In Figure 5B, we analyze the nature of these correlation effects, showing the same plot after z-score normalization that was performed separately for each concentration level (see section 2). Recall from Figures 2D,E that mean Euclidean distances (and also angles or cosine distances) differ between concentration levels, which complicates the comparison between concentration levels. Z-score normalization allows for a qualitative comparison of distances at different concentration levels in the sense that the effect of concentration-dependent feature (i.e., response) amplitude is removed, and with all concentration levels having the same mean distance. Distance changes on z-score normalized data are centered around zero with positive values indicating a change greater than the average and negative values indicating a smaller than average change.

Also after z-score normalization (Figure 5B), distance changes with rising concentration (x-axis) are correlated with both chemical distances (y-axis, Spearman's ρ = 0.43, *p* = 9.393×10^−7^) and distances at concentration level 10^−1^ (“color axis,” ρ = 0.82, *p* < 2.2 × 10^−16^). Thus, odor pairs that are chemically dissimilar tend to increase their response pattern distance with rising concentration, while chemically similar odor pairs tend to decrease their distance (relative to the concentration-specific baseline). The “color axis” in Figure 5B shows that, regardless of the relationship to chemical distances, there is an asymmetric distance development along the concentration axis. Odor pairs that are distant in response pattern space at 10^−1^ have increased their distance with rising concentration, while odor pairs that are close at 10^−1^ have decreased their distance (relative to baseline).

From the results of z-score normalization we can conclude that the observed distance changes are not due to concentration-dependent feature amplitude and baseline distances. Rather, distance changes are relative and can be observed on top of the general trend of increasing Euclidean distances with rising concentration. We note that a simple linear upscaling of the distances at 10^−4^ would be completely removed by z-score normalization. That is, the fact that the correlation effects persist after normalization is an indicator of non-linear distance changes along the concentration axis, introduced by additional and discriminatory glomerular responses.

For an explanation of the correlation between chemical distances and response distance changes along the concentration axis, consider the following argument: chemically similar odors should share many of their glomerular responses, while chemically dissimilar odors should elicit a larger number of non-matching responses. Consequently, adding further glomerular responses with rising concentration would recruit more non-matching, i.e., discriminating, responses for chemically dissimilar odors, and it would thus increase response patterns distances between chemically dissimilar odors more than distances between chemically similar odors, at least until saturation by too many (unspecific) glomerular responses is reached.

### 3.4. The effect of distance changes

In the previous section, we have visualized distance changes along the concentration axis. To assess whether these changes are in fact beneficial and whether they significantly improve representation of chemical (dis)similarity in odor response space, we performed Mantel tests (Mantel, 1967) for the correlation of distance matrices (see section 2).

**Figure 6:**
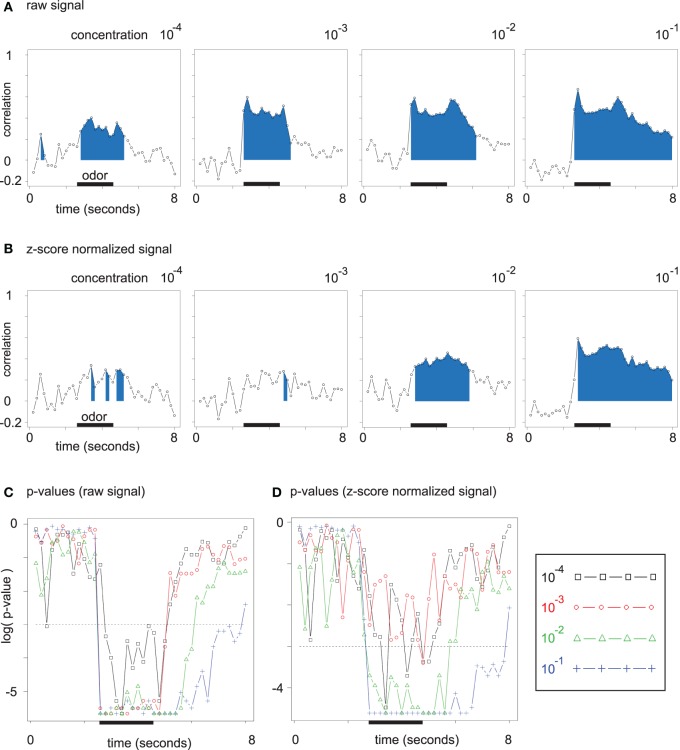
**(A)** Time-resolved correlation of the (Euclidean) chemical distance matrix and the Euclidean distance matrix of odor response patterns. The area under the curve is filled for all time points with significant (Mantel test) correlation. **(B)** Same plots as in **(A)**. Data was z-score normalized (see section 2). **(C)** Corrected *p*-values for the correlation analysis from **(A)** resolved over time. *P*-values are given on a log scale, and the threshold log(0.05) is marked with a dashed line. **(D)** Corrected *p*-values for the correlation analysis from **(B)**, i.e., for z-score normalized data.

We observed the correlation between the chemical distance matrix for odor molecules and the Euclidean distance matrix of odor response patterns. A high correlation (accompanied by a low *p*-value) indicates that odor representation is in close accordance with chemical (dis)similarity of the odors. We analyzed both Euclidean distances between unnormalized (Figure 6A) and normalized (Figure 6B) odor response patterns.

For each time point during the mean odor responses at all four concentration levels, we correlated the Euclidean distance matrix of odor response patterns with the chemical distance matrix of the corresponding odor molecules by performing Mantel tests. For all concentration levels in Figure 6A, the correlation rises during the interval of odor application. The development along the concentration axis from 10^−4^ to 10^−1^ leads to an increase in peak correlation between the two distance matrices. Furthermore, correlation remains elevated after odor offset for the higher odor concentrations, which reflects broader glomerular response curves with slower decay times.

As long as few glomeruli are active at low odor concentrations, regarding absolute response amplitude (on unnormalized data) is relevant for the correlation between the two Euclidean distance matrices. At higher odor concentrations, a larger number of discriminative features is available, such that qualitative distances (on normalized data) alone lead to a robust correlation (cp. Figures 6A,B).

While the correlations for unnormalized data in Figure 6A are due to both broadened response patterns and response amplitude increase, correlations for normalized data in Figure 6B are independent of absolute response amplitude and can be directly linked to the asymmetric distance changes observed in Figure 5B.

## 4. Discussion

The evidence gathered in this work suggests that (1) odor discrimination is improved at higher odor concentrations, as then response pattern distances are better estimates of chemical distances (Figure 6), and that (2) generalization over concentration levels of the same odor is possible to some extent due to pattern continuity and smooth transitions between patterns for concentration levels of the same odor (Figure 2).

Both, (1) and (2), are compatible with results from behavioral experiments: Pelz et al. (1997) found that odor discrimination performance of honeybees is increased for higher odor concentrations and that honeybees can generalize over concentration levels of the same odor. Note, though, that the honeybee's ability to generalize does not rule out its ability to discriminate between concentration levels if required for a learning task (Ditzen et al., 2003). In more recent studies, Wright and colleagues have confirmed that honeybees are better (Wright and Smith, 2004) and faster (Wright et al., 2009) at odor learning when odor concentration is high.

While we have not analyzed how odor information processing is actually performed in the honeybee brain, Yamagata et al. (2009) presented evidence for a system with two parallel pathways along which odor information is transported to the honeybee's higher-order brain centers, where the pathway via the medial antenno-protocerebral tract (m-APT) conveys information that is strongly influenced by odor concentration, whereas the lateral antenno-protocerebral tract (l-APT) conveys information that is more invariant with respect to odor concentration, while still maintaining information about concentration (Galizia and Rössler, 2010). This may be achieved by stronger gain control and lateral inhibition in the l-APT (Nawrot, 2012). The data analyzed in this paper is based on 17 glomeruli in the l-APT system and three glomeruli in the m-APT system (108, 135, 142; corresponding to T3-18, T3-45, T3-52).

Wachowiak et al. (2002) for turtles and Cleland et al. (2007) for rats proposed a normalization to the global olfactory bulb activity level, such that different concentration levels of the same odor remain similar. For *Drosophila*, Wang et al. (2003) found a global activity increase and a broadening of response patterns with increasing concentration. Friedrich et al. (1997) report similar results, increasing response strength and broadened patterns, for zebrafish. On the other hand, Stopfer et al. (2003) found changed patterns but no global activity increase over the concentration axis in the locust olfactory system. Common to all these observations (on PN/mitral cell data) is the finding that glomerular patterns do not change arbitrarily, but broaden with increasing concentration, a pattern continuity that likely makes different concentrations of the same odor cluster together, as explicitly stated in Stopfer et al. (2003), and as we have observed it.

Niessing and Friedrich (2010) suggested that large concentration differences between two samples of the same odor can lead to classification as two distinct odor objects on zebrafish data, but they also confirm that concentration levels of the same odor cluster together in response pattern space across a range of concentrations. In psychophysical/behavioral studies, Gross-Isseroff and Lancet (1988) for humans and Wright et al. (2005) for honeybees reported different perceptual qualities of identical odors given at different concentrations, suggesting that perfect generalization over arbitrarily large concentration ranges may not be possible. Likewise, our finding of improved odor coding at high concentrations implies also that there are limits to concentration level generalization as it should be easier to identify the odor at high than at low concentrations.

We note that all the bees used in this study were taken from the wild: they had various odor experiences, but no controlled experience regarding the 16 odors. Since odor experience, in particular when combined with reward as in appetitive conditioning trials, or when foraging for flower nectar, leads to a modification of odor-response patterns (Fernandez et al., 2009; Galizia and Szyszka, 2011), we would assume that if an animal was trained to either generalize across odor concentrations, or to differentiate between them, the distances shown here would either decrease, or increase, respectively.

Several lines of thought have been brought forward to explain invariance to odor concentration, at least over a certain range of concentrations. Uchida and Mainen (2007) have proposed that, if the odor is not monomolecular, concentration generalization may be achieved by comparing the concentration-invariant component ratio in an odor mixture, a strategy which could complement the generalization that is due to pattern continuity and stable distances. A model for how such component ratio recognition can be accomplished has been proposed by Zavada et al. (2011) for the macroglomerular complex of male moths.

Wilson and Mainen (2006) speculate that the temporal sequence of glomerular responses could be characteristic for the odor regardless of concentration, and concentration-invariant latency coding in insects has been suggested by Krofczik et al. (2009) and Belmabrouk et al. (2011). Our recordings presented here do not have the temporal resolution necessary to test how these temporal effects would add to the information already present in the combinatorial patterns at 5 Hz.

Another approach focuses on receptor neuron properties: Sandström (2010) developed a model for coding of odor concentration based on odor receptor neurons of the same type but with different concentration sensitivities that would, after processing at the glomerular level, render the identity of the responding mitral cells/PNs an approximate indicator of odor concentration. In our data we find that also the global activity level of the AL can be used as an approximate indicator for odor concentration. Multiglomerular PNs might relay this information to higher brain areas and use it in decoding odor information (Sachse and Galizia, 2006).

Apart from the concentration aspect, this paper also provides further evidence in the direction that the space of glomerular response patterns is a representation of chemical space. It has been observed before, both in vertebrates (Johnson et al., 2002) and invertebrates (Sachse et al., 1999), that glomerular response patterns cluster by a chemical feature, such as carbon chain length or functional group of the odor. The correlation analysis from Figure 6 generalizes this by utilizing a chemical distance metric (Haddad et al., 2008) that integrates a variety of features that all contribute to chemical (dis)similarity.

In a recent study on imaging data from the mouse olfactory bulb, Ma et al. (2012) also found that odors sharing a chemical feature cluster together in odor response pattern space. They came, however, to the conclusion that there is no correlation between chemical distances (Haddad et al., 2008) and odor response pattern distances. Methodological differences prevent a direct comparison to their study. In contrast to our paper, Ma et al. (2012) pooled data from different animals, and they concatenated odor responses patterns at different concentration levels, which were not the same as in this work, to form a hybrid response vector for the odor. This may overshadow significant correlations, considering the correlation changes along the concentration range (Figure 6).

We conclude that odor response patterns as recorded from PNs at the AL output reflect chemical (dis)similarity of the odors, that distances between (normalized) odor response patterns remain rather stable across concentration levels, and that, nevertheless, the correspondence to chemical distances is improved at higher odor concentrations. The latter is an aspect often overlooked in the literature that seems to be focused on the problem of concentration-invariant perception (Uchida and Mainen, 2007; Asahina et al., 2009; Schmuker et al., 2011; Cleland et al., 2012). Interestingly, though, Duchamp-Viret et al. (1990) noted that odor response patterns in the frog olfactory bulb broaden with rising concentration and that pattern separation improves at higher odor concentrations. Although they did not explicitly analyze distances and whether this is due to amplitude increase or to qualitative pattern changes, it is likely that similar results as reported here hold also for other organisms.

Returning to the question raised in the beginning, it appears that distance changes along the concentration axis are not a bug, but a feature, as they provide the basis for better odor perception at high concentrations. At the same time, generalization at least over a range of concentration levels remains possible because distances between spatial odor response patterns are not altered radically, but rather adjusted in a beneficial way. Given the structural similarities between olfactory systems, it is likely that the interplay of odor identity and concentration coding as described in this work is a general principle that can explain why odor discrimination is improved at higher odor concentrations.

### Conflict of interest statement

The authors declare that the research was conducted in the absence of any commercial or financial relationships that could be construed as a potential conflict of interest.
